# Drug Adverse Event Detection in Health Plan Data Using the Gamma Poisson Shrinker and Comparison to the Tree-based Scan Statistic

**DOI:** 10.3390/pharmaceutics5010179

**Published:** 2013-03-14

**Authors:** Jeffrey S. Brown, Kenneth R. Petronis, Andrew Bate, Fang Zhang, Inna Dashevsky, Martin Kulldorff, Taliser R. Avery, Robert L. Davis, K. Arnold Chan, Susan E. Andrade, Denise Boudreau, Margaret J. Gunter, Lisa Herrinton, Pamala A. Pawloski, Marsha A. Raebel, Douglas Roblin, David Smith, Robert Reynolds

**Affiliations:** 1 Department of Population Medicine, Harvard Medical School and Harvard Pilgrim Health Care Institute, 133 Brookline Avenue, 6th Floor, Boston, MA 02215, USA; E-Mails: fang_zhang@hphc.org (F.Z.); inna_dashevsky@hphc.org (I.D.); martin_kulldorff@hms.harvard.edu (M.K.); taliser_avery@hphc.org (T.R.A.); 2 The HMO Research Network Center for Education and Research in Therapeutics; E-Mails: robert.l.davis@kp.org (R.L.D.); susaneandrade@aol.com (S.E.A.); boudreau.d@ghc.org (D.B.); mjgunt@lcfresearch.org (M.J.G.); lisa.herrinton@kp.org (L.H.); pamala.a.pawloski@healthpartners.com (P.A.P.); marsha.a.raebel@kp.org (M.A.R.); douglas.roblin@kp.org (D.R.); david.h.smith@kpchr.org (D.S.); 3 Pfizer, Inc., New York, NY 10017, USA; E-Mail: kenneth.r.petronis@pfizer.com (K.R.P.); andrew.bate@pfizer.com (A.B.); robert.reynolds@pfizer.com (R.R.); 4 Kaiser Permanente Georgia, Atlanta, GA 30305, USA; 5 OptumInsight, Waltham, MA 02451, USA; E-Mail: arnold.chan@optum.com; 6 Harvard School of Public Health, Boston, MA 02115, USA; 7 Meyers Primary Care Institute, University of Massachusetts Medical School, the Meyers Primary Care Institute, Fallon Community Health Plan, Worcester, MA 01605, USA; 8 Center for Health Studies, Group Health Cooperative, Seattle, WA 98101, USA; 9 Lovelace Clinic Foundation, Albuquerque, NM 87106, USA; 10 Kaiser Permanente Northern California, Oakland, CA 94611, USA; 11 HealthPartners Research Foundation, Minneapolis, MN 55440, USA; 12 Kaiser Permanente Colorado, Denver, CO 80237, USA; 13 Kaiser Permanente Northwest, Portland OR 97227, USA

**Keywords:** pharmacovigilance, drug safety surveillance, adverse events data mining, gamma Poisson shrinkage, tree-based scan statistic

## Abstract

Background: Drug adverse event (AE) signal detection using the Gamma Poisson Shrinker (GPS) is commonly applied in spontaneous reporting. AE signal detection using large observational health plan databases can expand medication safety surveillance. Methods: Using data from nine health plans, we conducted a pilot study to evaluate the implementation and findings of the GPS approach for two antifungal drugs, terbinafine and itraconazole, and two diabetes drugs, pioglitazone and rosiglitazone. We evaluated 1676 diagnosis codes grouped into 183 different clinical concepts and four levels of granularity. Several signaling thresholds were assessed. GPS results were compared to findings from a companion study using the identical analytic dataset but an alternative statistical method—the tree-based scan statistic (TreeScan). Results: We identified 71 statistical signals across two signaling thresholds and two methods, including closely-related signals of overlapping diagnosis definitions. Initial review found that most signals represented known adverse drug reactions or confounding. About 31% of signals met the highest signaling threshold. Conclusions: The GPS method was successfully applied to observational health plan data in a distributed data environment as a drug safety data mining method. There was substantial concordance between the GPS and TreeScan approaches. Key method implementation decisions relate to defining exposures and outcomes and informed choice of signaling thresholds.

## 1. Introduction

Quantitative identification of unspecified medical product-adverse event (AE) relationships—often referred to as signal detection—is integral to worldwide medical product safety surveillance. Gamma Poisson Shrinkage (GPS) is a disproportionality method commonly applied to spontaneous reporting systems for signal detection [1]. Implementation of signal detection methods using routinely collected electronic data can expand the scope and scale of pharmacovigilance. In contrast with spontaneous reporting systems, however, little experience has been gained in the implementation and interpretation of GPS with observational electronic health care claims and administrative data. 

Investigators have proposed a variety of AE signal detection methods for observational data, including disproportionality approaches [1,2,3,4], the tree-based scan statistic (TreeScan) [5,6] and others [7,8,9,10]. Disproportionality approaches, including GPS, Information Component (IC) and the proportional reporting ratio (PRR) all have been applied to observational data, typically in two fundamentally different ways. One approach has been to apply the methods as closely as possible to their implementation in spontaneous report datasets by using observational data to mimic spontaneous reports of drug-event combinations [2,11], including the “spontaneous reporting system methods” of GPS, Information Component, PRR and reporting odds ratio (ROR) as implemented and evaluated in Schuemie *et al.* (2012). In one example, Curtis *et al.* (2008) identified exposure using the Medicare Current Beneficiary Survey (MCBS) and outcomes from a sample of medical claims linked to the MCBS. Monthly reports were created to mimic spontaneous reporting databases and analyzed as if they were spontaneous reports. Zorych (2011) used simulated and administrative claims data to evaluate disproportionality methods using three different approaches for creating the analytic 2 × 2 table; none accounted for exposed or unexposed person time. Schuemie (2011) used simulated data to conduct a pilot implementation of several modifications of GPS, comparing person-level and exposure-day level approaches for calculating observed and expected counts, and specifically adjusting for protopathic bias [3]. 

A second approach adapts these methods to try to better leverage the richness of longitudinal observational datasets. Noren *et al.* (2008; 2010) applied the Information Component Temporal Pattern Discovery (ICTPD) approach by comparing the observed count of a drug-outcome combination to an expected count based on general occurrences in the database, coupled with a self-controlled design element by comparing the Observed and Expected counts of an event after prescription to the Observed and Expected counts before prescription [4,12]. Schuemie *et al.* (2011) used simulated data to evaluate an alternative approach (Longitudinal GPS: LGPS) similar to our implementation here where rather than comparing to expected counts based on occurrence of events for patients taking other prescribed products he utilizes exposed and non-exposed time at risk to develop a richer denominator [13]. 

Both implementation approaches have strengths and weaknesses. The LGPS method computes expected counts of medical events during drug exposure based on an aggregate of unexposed patient time in ever‐exposed and unexposed patients, potentially introducing confounding as unexposed patients may be less likely to have events related to the drug indication or underlying disease than the exposed population. For ICTPD, one of the two comparisons is of events occurring within a specific time after a dispensing of the drug of interest to all observations of that event after exposure to all other drugs but within the same at-risk period to give an Expected count. Inclusion of drugs associated with the outcome of interest will inflate the Expected count, and could lead to a reduced ICTPD score for the drug-event of interest; the inverse could occur with protective effects [14,15,16]. The GPS and ICTPD approaches and others differ in how a score is derived for the drug-outcome pairs, but also in terms of the test statistic, the choice of signaling threshold, as well as differences in implementation, some of which reflect the differences in the observational databases used (e.g., different terminological classifications of outcomes) [17].

More recently, Schuemie *et al.* (2012) and Ryan *et al.* (2012) published a comparison of multiple signal detection methods using longitudinal data across three countries [13,18]. The approaches are similar. Schuemie (2012) compared 10 methods using a set of positive and negative controls (drug-event pairs) for comparison. They reported positive results for most methods, including LGPS. Direct applicability of their results to routine open ended signal detection is hard to assess as they limited their assessment to a small set of known associations and their comparisons were based on area under the curve estimates on ROC curves where all sensitivity and specificity thresholds are considered equally important. In practice the tail ends of ROC curves may not be appropriate to consider in assessing surveillance approach effectiveness; if there is great differential performance between methodological approaches in these tails of ROC curves a misleading impression of performance and erroneous comparisons between approaches can be made. Schuemie *et al.* (2012) focused on point estimates instead of the lower thresholds of confidence limits that are more commonly used in signal detection to protect against spurious findings [1]. Finally, focusing on point estimates creates the potential to favor methods that routinely over-estimate risk.

Given that the GPS approach has shown promising results for use in longitudinal data [2,3,5,11,13], we furthered the prior work by applying GPS in a “real-world” environment not limited to specific associations but rather including non-prespecified drug-event pairs for evaluation. Such a real-world open ended discovery approach has not to our knowledge been taken with a GPS based method, although open ended discovery was done in Noren *et al.* (2010) for the ICTPD approach [12]. Our implementation closely mimicked the approach described in the U.S. Food and Drug Administration’s (FDA) Mini-Sentinel project for evaluation of non-prespecified AEs [19]. 

We present a pilot study evaluating the implementation of GPS for drug-AE signal detection using routinely-collected electronic medical encounter data in a multi-site environment. We also compare the GPS results to findings from a TreeScan study that used identical input datasets. 

## 2. Methods

### 2.1. Overview

Signal detection using observational data requires three key specifications: (i) the analytic approach related to calculating exposures, identifying cases, defining comparators, and handling censoring; (ii) the statistical method used; and (iii) the signaling thresholds. Our implementation compared the rate of exposed outcomes with an expected count based on unexposed time. Therefore, the specific question was whether there is a statistical signal of excess risk of an outcome during exposed time as compared to unexposed time. In this paper we define a “signal” as a statistical association between a drug and a diagnosis within an exploratory framework without any requirement for verification of case status by medical record review or other confirmatory analysis. These “statistical signals” do not imply causality, but rather represent an association that meets pre-specified signaling thresholds that may warrant further investigation. Statistical signals identified using signal detection methods often can be explained by bias and confounding. We focused on signal detection implementation approaches using observational data, not prioritization and investigation of the signals identified. Such signal refinement requires additional methods and dedicated resources beyond the scope of this study [20].

### 2.2. Data and Study Population

The study cohort consisted of approximately 3.4 million privately-insured health plan members enrolled between 1999 and 2003 distributed roughly equally across the nine plans in the HMO Research Network Center for Education and Research on Therapeutics: Harvard Pilgrim Health Care, Kaiser Permanente Georgia, Meyers Primary Care Institute, Group Health Cooperative, Lovelace Clinic Foundation, Kaiser Permanente Northern California, HealthPartners Research Foundation, Kaiser Permanente Colorado, and Kaiser Permanente Northwest. Each health plan maintains an electronic database of member demographics, enrollment, outpatient pharmacy dispensing, and inpatient and outpatient encounters. These data have been used in several drug safety studies [21,22,23,24,25,26,27] and described in detail elsewhere [21,22,28].

Demographic information includes date of birth and sex. Enrollment consists of enrollment start and stop dates and a drug coverage indicator. Pharmacy dispensing data includes dispensing date, national drug code, units dispensed, and days supplied. Encounter information includes all diagnosis codes recorded during ambulatory and inpatient encounters. 

We employed a distributed data model [29,30,31] that enabled sites to share only summarized count information for aggregation and analysis. The study was approved by the Institutional Review Board at each site. 

### 2.3. Study Drugs

We identified users of two antifungal drugs, terbinafine and itraconazole, and two diabetes drugs, pioglitazone and rosiglitazone. These products were selected because they have substantial exposure, well-characterized risks, and allow for within-indication comparisons. We noted established associations between terbinafine and itraconazole and risk of liver disease [32,33] and allergic reaction [34,35]. Itraconazole and both diabetes drugs carry black box warnings for congestive heart failure on the U.S. FDA approved product labeling . Each drug was analyzed separately, without consideration of prior or concurrent exposures. 

### 2.4. Diagnosis Definitions

Starting with all ICD-9-CM diagnosis codes we removed diagnosis codes associated with conditions unlikely to be drug-associated acute AEs (e.g., neoplasms, pregnancy and perinatal conditions, congenital anomalies, injuries and poisoning, diabetes). The remaining 1676 diagnosis codes were grouped using the Multi-level Clinical Classifications Software (MLCCS) [36]. The MLCCS is a hierarchical system with four levels of clinical concepts denoted by four 2-digit identifiers. The top level MLCCS identifies 18 body systems, and each can have up to three sublevels, as represented by the 2nd, 3rd, and 4th 2-digit codes. Each diagnosis code belongs to one classification group at each level of the MLCCS system, creating a hierarchical tree structure, where related diagnoses are close to each other on the tree. The exclusion process resulted in 183 overlapping groupings of related clinical concepts that were evaluated as potential AEs. Table 1 illustrates the hierarchical tree structure. Analyses were done at all four levels of granularity separately. Since we created a single set of diagnoses across products, we expected to identify some “signals” that represent bias and confounding common to uncontrolled observational studies (e.g., pioglitazone patients will have a higher rate of diabetes related complications such as eye disorders).

### 2.5. Contributed Person Time

**Figure 1:**
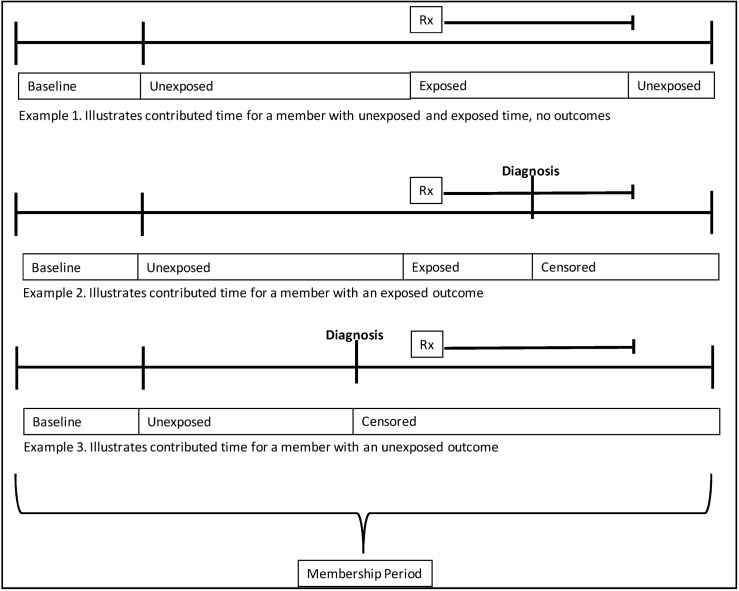
Contributed person time: member timelines.

All individuals with a membership period with medical and drug coverage over 180 days contributed person time. Membership gaps of 60 days or less were bridged to create continuous membership periods. Contributed days began after a 180 day baseline period, and ended for that member at the first incident diagnosis of any clinical concept, the last day of enrollment, or the end of the study period (December 31, 2003), whichever came first. Figure 1 illustrates how contributed time was parsed. The baseline period was used as to identify prior diagnoses; no exclusions were applied during baseline. 

**Table 1:** A small subset of the Multi-Level Clinical Classification Tree with International Classification of Diseases, Ninth Revision (ICD-9) codes associated with a specific level.

07	Diseases of the Circulatory System
07.01	Hypertension
07.01.02	Hypertension with Complications and Secondary Hypertension
07.01.02.01	Hypertensive Heart and/or Renal Disease (402.00–404.93)
07.01.02.02	Other Hypertensive Complications (405.01–405.99,437.2)
07.02	Diseases of The Heart
07.02.01	Heart Valve Disorders
07.02.01.02	Nonrheumatic Mitral Valve Disorders (424.0)
07.02.01.03	Nonrheumatic Aortic Valve Disorders (424.1)
07.02.01.04	Other Heart Valve Disorders (424.2, 424.3, 785.2, 785.3)
07.02.02	Peri; Endo; and Myocarditis; Cardiomyopathy (Except that Caused by TB or STD)
07.02.02.01	Cardiomyopathy (425.0–425.9)
07.02.03	Acute Myocardial Infarction (410.0–410.92)
07.02.04	Coronary Atherosclerosis and Other Heart Disease
07.02.04.01	Angina Pectoris (413.0–413.9)
07.02.04.02	Unstable Angina (Intermediate Coronary Syndrome) (411.1)
07.02.04.03	Other Acute and Subacute Forms of Ischemic Heart Disease (411.0, 411.8–411.89)
07.02.04.04	Coronary Atherosclerosis (414.05)
07.02.04.05	Other Forms of Chronic Heart Disease (414.8, 414.9)
07.02.04.00	Other (414.06)

### 2.6. Drug Exposure

Contributed days were either exposed or unexposed person time. Treatment episodes (*i.e.*, exposed person time) began the day after a drug dispensing and continued until the end of exposure based on days supplied. Consecutive dispensings were combined, and exposure gaps of six days or less were bridged to create continuous episodes. Unexposed person time was defined as all contributed days without exposure. For each product we calculated total exposed and unexposed person time.

### 2.7. Outcomes

We defined an incident outcome as the first observed diagnosis during contributed time that was not observed during baseline. Only the first incident outcome observed was counted and designated as exposed or unexposed; this restriction is necessary for the TreeScan analysis that adjusts for multiple testing and was applied here to enable comparison across methods. 

### 2.8. Calculation of Observed and Expected Counts

Exposed outcomes are the number of incident outcomes observed during exposed days. The unadjusted expected count is the number of exposed days times the rate of incident outcomes during unexposed days, calculated as the number of unexposed outcomes divided by the number of unexposed days. Using indirect standardization, we adjusted expected counts for age, sex, and health plan. 

Following the distributed data model approach [29,30,31], each site executed analytic code provided by the study coordinating center. Analytic program output contained counts of exposed and unexposed days and outcomes by age (5-year strata) and sex; counts were transferred to the coordinating center for aggregation and analysis. 

### 2.9. Gamma Poisson Shrinker

The GPS was proposed by DuMouchel [37,38] as a signal detection tool for large frequency tables with both observed (O) and expected (E) counts for each drug-outcome pair. It assumes the observed count of any drug-outcome pair follows the Poisson distribution. For spontaneous reports, there are no drug exposure denominator data, so the expected counts are calculated under the null assumption that each drug has the same proportion of diagnosis codes. That is, the expected counts are internally derived assuming the independence of drug and event reporting, and calculated as the product of two marginal frequencies of the drug-outcome pair and the total count of all observed events. For example, if seizures comprise 1% of all the diagnosis codes, over all drugs, and itraconazole has a total of 800 diagnoses, then the expected number of seizures is eight for itraconazole. 

Unlike spontaneous reporting databases, population-based event monitoring using health plan data can calculate observed and expected counts based on observed exposure information and diagnoses observed during exposed and unexposed time. GPS can be directly adapted to such settings with the internally derived expected counts replaced by the expected counts constructed using the denominators. 

Details of the GPS algorithm have been extensively described [37,38,39,40]. Briefly, for each drug-outcome pair, the primary parameter of interest was the risk ratio. Rather than using the observed over expected (O/E), GPS uses the empirical Bayesian geometric mean (EBGM) posterior distribution of the risk ratio and the surrounding confidence interval for each drug-outcome pair to identify statistical signals of excess risk. To prevent spurious false positives due to implausibly high risk ratios, GPS implements a Bayesian framework that “shrinks” O/E estimates towards a value which is close to the average O/E values for all drug-event pairs at each level of granularity. For these data, that average is about 1.5. GPS accomplishes this by use of an empirical Bayesian framework where the values of all O/E estimates are modeled as a mixture distribution. This so-called “prior distribution” is then combined with data on a specific drug–outcome pair to give a score: the EBGM. Further work would be needed to determine whether shrinkage towards an average value far from one is justified or represents an artificial attribute that might adversely impact performance of the GPS approach. We evaluated each level of the diagnosis tree separately. 

We used two signaling thresholds for GPS. The first is the lower bound of the 95% posterior probability interval of 1.5 or more (medium threshold). Since the average O/E for our population was close to 1.5, this threshold mimics an excess risk but is not adjusted for multiple testing. To informally adjust for multiple testing when applying data mining approaches to spontaneous reporting, the U.S. FDA uses the lower bound of the 90% posterior probability interval of EBGM of greater or equal to two as the signal threshold for their spontaneous reporting system [41]. We used this threshold as our most stringent signaling criteria. 

### 2.10. Comparison to Tree-Based Scan Statistic

TreeScan is a signal detection method that simultaneously looks for excess risk in any of a large number of individual cells in a database and in groups of closely related cells, formally adjusting the *p*-values for the multiple testing inherent in the large number of overlapping diagnosis groups evaluated [6,42,43]. The paper by Kulldorff *et al.* (2012) details the TreeScan approach for drug safety surveillance [42]. In brief, a hierarchical classification tree is first constructed for the outcomes where related diagnoses are close to each other on the tree. Different cuts on the tree are then made, and it is evaluated whether the group of diagnoses on that branch of the tree has an excess risk of occurring among the drug users. In this way, the method evaluates both very specific outcome definitions such as Paralytic Ileus (a single leaf on the tree) as well as large groups of related outcomes such as Diseases of the Digestive System (one of the largest branches on the tree). The method formally adjusts for the multiple testing inherent in the hundreds or thousands of different cuts evaluated. 

For the comparison between GPS and TreeScan we used identical input datasets of age and sex stratified O and E counts for each MLCCS node separately for each drug. We conducted a *post-hoc* comparison of the GPS and TreeScan results focusing on differences in the number of signals identified overall and by signaling threshold. For this comparison we define two signaling thresholds for TreeScan: multiple testing adjusted *p*-values of <0.001 and 0.001 < *p* < 0.05. This comparison was made to help put the GPS results in context using a different statistical signaling method with the same input datasets. We used the product label, medical literature, and clinician input to informally categorize the signals as known, likely confounded, or previously unknown. Formal signal evaluation was beyond the scope of the project.

## 3. Results

### 3.1. Terbinafine and Itraconazole

Across all thresholds and nodes on the classification tree we identified 10 GPS terbinafine signals and four itraconazole signals (Table 2). One of the 10 terbinafine signals met the highest GPS threshold and five met the highest TreeScan threshold. Of the four itraconazole signals, one met the highest GPS threshold and two met the highest TreeScan threshold. The antifungal signals represent known AEs (e.g., liver conditions, allergic reactions, nausea) or likely confounding by indication (e.g., skin and subcutaneous tissue diagnoses) [32,44,45], Over 46,000 exposed days for terbinafine, 415,000 exposed days for itraconazole, and 1.1 billion unexposed days were assessed.

### 3.2. Pioglitazone and Rosiglitazone

Of the 35 pioglitazone signals identified by either method, 15 met the highest GPS signaling threshold and 27 met the highest TreeScan threshold. For the 22 rosiglitazone signals, six met the highest GPS threshold and 15 met the highest TreeScan threshold (Table 3). Most of pioglitazone signals were in four body systems, including signals for Coronary Atherosclerosis and Other Heart Disease (MLCCS node 07.02.04), Congestive Heart Failure (07.02.11 and 07.02.11.01), and Peripheral and Visceral Atherosclerosis (07.04.01). Pioglitazone also had high threshold GPS signals for Nephritis, Nephrosis and Renal Sclerosis (10.01.01) and Chronic Renal Failure (10.01.03). Over 1.3 million exposed days for pioglitazone, 637,000 exposed days for rosiglitazone, and 1.1 billion unexposed days were assessed.

The cardiovascular and renal signals are known AEs or likely due to confounding. For example, diabetes patients have a higher risk for renal impairment and, since diabetes medications such as metformin and glyburide are contraindicated for those with renal dysfunction, diabetes patients with renal impairment may have been channeled to pioglitazone and rosiglitazone. Both drugs also signaled strongly for chronic ulcer of the skin, and pioglitazone signaled for eye disorders, another likely example of confounding. 

**Table 2:** Results for terbinafine and itraconazole.

MLCCS	Diagnosis	Terbinafine						Itraconazole					
		Obs	Exp	O/E	EBGM	GPS Signal	TreeScan *p*-value	Obs	Exp	O/E	EBGM	GPS Signal	TreeScan *p*-value
05	Mental Disorders	0	0.6	0.0	1.3		.	0	0.1	0.0	1.4		.
06	Diseases Of The Nervous System And Sense Organs	37	22.7	1.6	1.6		0.28	11	5.2	2.1	1.7		0.54
07	Diseases Of The Circulatory System	51	44.4	1.1	1.2		.	21	10.2	2.1	1.7		0.13
07.01	Hypertension	1	2.0	0.5	1.3		.	1	0.6	1.8	1.5		.
07.02	Diseases Of The Heart	24	21.4	1.1	1.2		.	9	5.0	1.8	1.6		.
07.02.01	Heart Valve Disorders	1	2.8	0.4	1.2		.	0	0.7	0.0	1.3		.
07.02.03	Acute Myocardial Infarction	0	0.8	0.0	1.3		.	1	0.2	4.7	1.7		.
07.02.04	Coronary Atherosclerosis And Other Heart Disease	3	3.4	0.9	1.3		.	1	0.9	1.1	1.5		.
07.02.07	Other And Ill-Defined Heart Disease	3	1.0	3.1	1.7		.	3	0.2	12.3	3.9		0.09
07.02.08	Conduction Disorders	1	0.5	1.9	1.5		.	1	0.1	7.9	1.8		.
07.02.09	Cardiac Dysrhythmias	16	10.1	1.6	1.5		.	3	2.2	1.4	1.5		.
07.03	Cerebrovascular Disease	4	2.9	1.4	1.5		.	2	0.7	2.9	1.6		.
07.04	Diseases Of Arteries; Arterioles; And Capillaries	17	13.8	1.2	1.3		.	5	3.0	1.7	1.5		.
07.05	Diseases Of Veins And Lymphatics	5	4.3	1.2	1.4		.	4	0.9	4.4	1.9		0.47
09	Diseases Of The Digestive System	63	37.2	1.7	1.6		0.007	15	8.2	1.8	1.6		0.63
09.03	Diseases Of Mouth; Excluding Dental	5	3.3	1.5	1.5		.	0	0.7	0.0	1.3		.
09.04	Upper Gastrointestinal Disorders	8	7.2	1.1	1.3		.	5	1.5	3.3	1.8		0.53
09.06	Lower Gastrointestinal Disorders	1	0.8	1.3	1.5		.	1	0.2	5.8	1.7		.
09.07	Biliary Tract Disease	2	1.6	1.3	1.4		.	1	0.3	3.1	1.6		.
09.08	Liver Disease	14	3.1	4.5	3.5	**	0.00005	1	0.7	1.4	1.5		.
09.08.02	Other Liver Diseases	14	3.1	4.5	3.37	**	0.00005	1	0.7	1.4	1.5		.
09.08.02.04	Other And Unspecified Liver Disorders	14	2.8	5.1	4.1	***	0.00002	1	0.6	1.6	1.5		.
09.09	Pancreatic Disorders (Not Diabetes)	2	0.3	5.9	1.9		.	1	0.1	15.0	2.0		.
09.09.03	Other Pancreatic Disorders	2	0.1	36.9	4.2		0.06	1	0.0	69.3	2.2		.
09.10	Gastrointestinal Hemorrhage	12	6.4	1.9	1.6		.	2	1.5	1.4	1.5		.
09.12	Other Gastrointestinal Disorders	19	14.4	1.3	1.4		.	4	3.3	1.2	1.4		.
10	Diseases Of The Genitourinary System	29	23.5	1.2	1.3		.	1	5.5	0.2	0.4		.
11	Complications Of Pregnancy; Childbirth; And The Puerperium	0	0.6	0.0	1.2		.	1	0.1	8.1	1.7		.
12	Diseases Of The Skin And Subcutaneous Tissue	125	51.6	2.4	2.2	**	0.00001	31	11.2	2.8	2.1	**	0.00001
12.01	Skin And Subcutaneous Tissue Infections	4	3.7	1.1	1.4		.	3	0.9	3.5	1.7		0.84
12.02	Other Inflammatory Condition Of Skin	25	10.6	2.4	1.9		0.010	9	2.1	4.2	2.4		0.02
12.03	Chronic Ulcer Of Skin	1	0.3	3.6	1.6		.	0	0.1	0.0	1.5		.
12.04	Other Skin Disorders	95	37.0	2.6	2.3	**	0.00001	19	8.1	2.3	1.8		0.05
13	Diseases Of The Musculoskeletal System And Connective Tissue	60	43.3	1.4	1.4		0.59	15	9.1	1.7	1.6		0.84
13.01	Infective Arthritis And Osteomyelitis (Except That Caused By TB Or STD	1	0.3	3.6	1.6		.	4	0.1	56.8	19.5	***	0.00001
13.08	Other Connective Tissue Disease	59	42.7	1.4	1.4		0.63	11	8.9	1.2	1.4		.
16	Injury And Poisoning	2	0.8	2.6	1.6		.	0	0.2	0.0	1.4		.
17	Symptoms; Signs; And Ill-Defined Conditions And Factors Influencing Health	62	38.1	1.6	1.6		0.02	15	8.5	1.8	1.6		0.75
17.01	Symptoms; Signs; And Ill-Defined Conditions	62	38.1	1.6	1.6		0.02	15	8.5	1.8	1.6		0.75
17.01.01	Syncope	3	2.3	1.3	1.4		.	1	0.5	1.9	1.5		.
17.01.06	Nausea And Vomiting	10	3.9	2.6	1.9		0.42	6	0.9	6.4	3.5		0.03
17.01.07	Abdominal Pain	21	18.1	1.2	1.3		.	2	4.1	0.5	1.1		.
17.01.08	Malaise And Fatigue	3	2.9	1.0	1.4		.	1	0.7	1.5	1.5		.
17.01.09	Allergic Reactions	25	10.8	2.3	2.0		0.01	5	2.2	2.3	1.7		.

MLCCS = Multi-level Clinical Classifications System; Obs = Observed; Exp = Expected; O/E = Observed/Expected; TreeScan *p* = multiple testing adjusted *p*-values, *p* > 0.90 is indicated with ‘.’; EBGM: empirical Bayesian geometric mean; *** GPS Signal at lower 90% CI bound ≥2; ** Signal at lower 95% CI bound >1.5; Table includes (1) all major disease category headings; (2) any disease categories that signaled for GPS or with a *p*-value ≤ 0.10; (3) any parents/grandparent of categories that signaled, and (4) any sibling of a disease category that signaled as long as there were observed events. We excluded any categories that were exactly the same as the parent.

**Table 3:** Results for pioglitazone and rosiglitazone.

MLCCS	Diagnosis	Pioglitazone						Rosiglitazone					
		Obs	Exp	O/E	EBGM	GPS Signal	TreeScan *p*-value	Obs	Exp	O/E	EBGM	GPS Signal	TreeScan *p*-value
05	Mental Disorders	4	1.7	2.4	1.6		.	1	0.9	1.1	1.5		.
06	Diseases Of The Nervous System And Sense Organs	197	90.7	2.2	2.1	**	0.00001	75	45.1	1.7	1.6		0.003
06.03	Paralysis	2	1.1	1.9	1.5		.	0	0.7	0.0	1.3		.
06.04	Epilepsy; Convulsions	3	4.0	0.8	1.3		.	2	2.3	0.9	1.4		.
06.05	Headache; Including Migraine	4	11.6	0.3	0.6		.	5	5.6	0.9	1.3		.
06.06	Coma; Stupor; And Brain Damage	3	1.3	2.4	1.6		.	1	0.6	1.7	1.5		.
06.07	Eye Disorders	185	72.9	2.5	2.4	***	0.00001	67	35.9	1.9	1.8		0.00004
06.07.01	Cataract	123	51.4	2.4	2.3	**	0.00001	47	24.2	1.9	1.8		0.002
06.07.03	Glaucoma	62	21.4	2.9	2.6	***	0.00001	20	11.7	1.7	1.6		0.80
07	Diseases Of The Circulatory System	378	177.7	2.1	2.1	**	0.00001	186	94.8	2.0	1.9	**	0.00001
07.01	Hypertension	34	16.3	2.1	1.8		0.009	4	3.1	1.3	1.4		.
07.01.02	Hypertension With Complications And Secondary Hypertension	34	16.3	2.1	1.9		0.009	4	3.1	1.3	1.4		.
07.01.02.01	Hypertensive Heart And/Or Renal Disease	34	16.1	2.1	1.9		0.007	4	2.9	1.4	1.5		.
07.02	Diseases Of The Heart	190	86.6	2.2	2.1	**	0.00001	116	51.9	2.2	2.1	**	0.00001
07.02.01	Heart Valve Disorders	15	10.1	1.5	1.5		.	14	6.7	2.1	1.8		0.54
07.02.02	Peri-; Endo-; And Myocarditis; Cardiomyopathy (Except That Caused	6	2.2	2.7	1.8		0.89	4	1.1	3.8	1.9		0.80
07.02.03	Acute Myocardial Infarction	12	4.0	3.0	2.0		0.08	5	1.8	2.8	1.8		.
07.02.04	Coronary Atherosclerosis And Other Heart Disease	51	18.2	2.8	2.4	**	0.00001	24	10.0	2.4	2.0		0.01
07.02.04.00	Coronary Atherosclerosis And Other Heart Disease	11	3.6	3.1	2.1		0.10	3	1.6	1.8	1.6		.
07.02.04.01	Angina Pectoris	16	6.3	2.5	2.0		0.08	8	3.1	2.6	1.8		0.66
07.02.04.02	Unstable Angina (Intermediate Coronary Syndrome)	5	2.0	2.5	1.7		.	6	1.5	4.1	2.2		0.26
07.02.04.03	Other Acute And Subacute Forms Of Ischemic Heart Disease	4	1.0	4.2	2.0		0.70	1	0.3	2.9	1.6		.
07.02.04.05	Other Forms Of Chronic Heart Disease	15	5.2	2.9	2.1		0.03	6	3.4	1.8	1.6		.
07.02.05	Nonspecific Chest Pain	2	1.8	1.1	1.4		.	2	1.6	1.3	1.5		.
07.02.06	Pulmonary Heart Disease	1	1.2	0.8	1.4		.	4	0.8	5.0	2.2		0.48
07.02.07	Other And Ill-Defined Heart Disease	5	5.2	1.0	1.3		.	11	3.1	3.5	2.2		0.03
07.02.08	Conduction Disorders	2	2.2	0.9	1.4		.	4	1.4	2.8	1.7		.
07.02.09	Cardiac Dysrhythmias	48	33.3	1.4	1.5		0.63	28	21.9	1.3	1.3		.
07.02.10	Cardiac Arrest And Ventricular Fibrillation	4	0.9	4.4	2.0		0.62	0	0.4	0.0	1.4		.
07.02.11	Congestive Heart Failure; Nonhypertensive	44	7.3	6.0	5.9	***	0.00001	20	3.2	6.3	6.0	***	0.00001
07.02.11.00	Congestive Heart Failure; Nonhypertensive	4	0.1	54.6	14.1	***	0.00002	1	0.0	26.4	2.1		.
07.02.11.01	Congestive Heart Failure	35	6.3	5.5	5.4	***	0.00001	16	2.7	5.8	5.3	***	0.00002
07.02.11.02	Heart Failure	5	0.9	5.4	2.5		0.19	3	0.4	7.3	2.5		0.43
07.03	Cerebrovascular Disease	40	15.1	2.6	2.2	**	0.00001	13	7.1	1.8	1.6		.
07.03.01	Acute Cerebrovascular Disease	14	5.9	2.4	1.9		0.27	1	2.3	0.4	1.2		.
07.03.02	Occlusion Or Stenosis Of Precerebral Arteries	7	2.8	2.5	1.8		0.86	8	1.3	6.1	4.1	**	0.00472
07.03.03	Other And Ill-Defined Cerebrovascular Disease	3	1.6	1.9	1.6		.	1	1.2	0.8	1.4		.
07.03.04	Transient Cerebral Ischemia	14	3.9	3.6	2.4		0.006	2	1.6	1.3	1.5		.
07.03.05	Late Effects Of Cerebrovascular Disease	2	0.9	2.2	1.6		.	1	0.7	1.5	1.5		.
07.04	Diseases Of Arteries; Arterioles; And Capillaries	81	44.8	1.8	1.7		0.00002	41	25.7	1.6	1.6		0.28149
07.04.01	Peripheral And Visceral Atherosclerosis	33	7.6	4.4	4.1	***	0.00001	18	3.8	4.7	4.0	***	0.00002
07.04.01.01	Atherosclerosis Of Arteries Of Extremities	9	1.6	5.5	3.6	**	0.005	3	0.7	4.2	1.9		.
07.04.01.02	Peripheral Vascular Disease Unspecified	21	4.9	4.3	3.5	***	0.00001	11	2.4	4.7	3.1	**	0.0023
07.04.01.03	Other Peripheral And Visceral Atherosclerosis	3	1.0	3.0	1.7		.	4	0.8	5.2	2.2		0.43304
07.04.02	Aortic; Peripheral; And Visceral Artery Aneurysms	1	1.9	0.5	1.3		.	3	1.1	2.7	1.7		.
07.04.03	Aortic And Peripheral Arterial Embolism Or Thrombosis	5	0.9	5.3	2.5		0.20	2	0.4	4.6	1.8		.
07.04.04	Other Circulatory Disease	42	34.4	1.2	1.3		.	18	20.4	0.9	1.1		.
07.04.04.01	Hypotension	8	1.8	4.6	2.6		0.04	0	0.7	0.0	1.4		.
07.04.04.02	Other And Unspecified Circulatory Disease	34	32.7	1.0	1.1		.	18	19.6	0.9	1.1		.
07.05	Diseases Of Veins And Lymphatics	33	14.9	2.2	1.9		0.004	12	6.9	1.7	1.6		.
07.05.01	Phlebitis; Thrombophlebitis And Thromboembolism	9	4.2	2.1	1.7		.	8	2.5	3.2	2.0		0.27
07.05.02	Varicose Veins Of Lower Extremity	5	5.8	0.9	1.2		.	1	2.7	0.4	1.2		.
07.05.04	Other Diseases Of Veins And Lymphatics	19	5.0	3.8	2.9	**	0.00002	3	1.7	1.7	1.5		.
09	Diseases Of The Digestive System	131	116.4	1.1	1.2		.	80	62.0	1.3	1.3		0.79
10	Diseases Of The Genitourinary System	186	75.0	2.5	2.3	***	0.00001	96	45.4	2.1	2.0	**	0.00001
10.01	Diseases Of The Urinary System	167	63.7	2.6	2.5	***	0.00001	86	35.5	2.4	2.2	**	0.00001
10.01.01	Nephritis; Nephrosis; Renal Sclerosis	28	1.2	24.3	21.0	***	0.00001	6	0.7	8.7	5.7	**	0.005
10.01.02	Acute And Unspecified Renal Failure	3	1.2	2.6	1.7		.	1	0.6	1.6	1.5		.
10.01.03	Chronic Renal Failure	14	1.6	9.0	8.3	***	0.00001	7	0.7	10.1	7.8	***	0.0001
10.01.04	Urinary Tract Infections	6	1.3	4.7	2.4		0.15	2	0.6	3.4	1.7		.
10.01.05	Calculus Of Urinary Tract	6	8.6	0.7	1.1		.	3	4.2	0.7	1.2		.
10.01.06	Other Diseases Of Kidney And Ureters	30	6.2	4.8	4.7	***	0.00001	14	3.4	4.1	2.9	**	0.0004
10.01.06.02	Other And Unspecified Diseases Of Kidney And Ureters	30	5.5	5.5	5.3	***	0.00001	14	3.0	4.7	3.6	**	0.00004
10.01.07	Other Diseases Of Bladder And Urethra	0	1.0	0.0	1.3		.	1	0.6	1.8	1.5		.
10.01.08	Genitourinary Symptoms And Ill-Defined Conditions	80	42.8	1.9	1.8		0.00002	52	24.7	2.1	1.9	**	0.00002
10.01.08.01	Hematuria	13	11.9	1.1	1.3		.	14	7.5	1.9	1.7		0.86
10.01.08.02	Retention Of Urine	3	2.8	1.1	1.4		.	1	1.3	0.7	1.4		.
10.01.08.03	Other And Unspecified Genitourinary Symptoms	64	28.1	2.3	2.1	**	0.00001	37	15.9	2.3	2.1	**	0.00007
10.03	Diseases Of Female Genital Organs	19	11.2	1.7	1.6		0.88	10	9.9	1.0	1.3		.
11	Complications Of Pregnancy; Childbirth; And The Puerperium	0	0.5	0.0	1.3		.	0	0.3	0.0	1.3		.
12	Diseases Of The Skin And Subcutaneous Tissue	205	166.6	1.2	1.3		0.23	78	70.5	1.1	1.2		.
12.01	Skin And Subcutaneous Tissue Infections	17	7.9	2.2	1.7		0.26	9	4.2	2.1	1.6		.
12.02	Other Inflammatory Condition Of Skin	41	33.8	1.2	1.3		.	15	13.1	1.1	1.3		.
12.03	Chronic Ulcer Of Skin	13	1.1	11.8	10.7	***	0.00001	9	0.4	21.2	16.4	***	0.00001
12.03.02	Chronic Ulcer Of Leg Or Foot	13	1.1	11.8	10.4	***	0.00001	9	0.4	21.2	15.1	***	0.00001
12.04	Other Skin Disorders	134	123.8	1.1	1.1		.	45	52.7	0.9	1.0		.
13	Diseases Of The Musculoskeletal System And Connective Tissue	187	131.8	1.4	1.4		0.00008	84	57.7	1.5	1.5		0.07
13.01	Infective Arthritis And Osteomyelitis (Except That Caused By TB Or STD	2	1.0	2.0	1.5		.	1	0.6	1.6	1.5		.
13.07	Systemic Lupus Erythematosus And Connective Tissue Disorders	1	1.1	0.9	1.4		.	2	0.7	2.9	1.6		.
13.08	Other Connective Tissue Disease	184	129.6	1.4	1.4		0.0001	81	56.4	1.4	1.4		0.13
16	Injury And Poisoning	2	1.8	1.1	1.4		.	1	0.9	1.1	1.4		.
17	Symptoms; Signs; And Ill-Defined Conditions And Factors Influencing Health	128	106.8	1.2	1.2		.	57	50.4	1.1	1.2		.

MLCCS = Multi-level Clinical Classifications System; Obs = Observed; Exp = Expected; O/E = Observed/Expected; TreeScan *p* = multiple testing adjusted *p*-values, *p* > 0.90 is indicated with ‘.’; EBGM: empirical Bayesian geometric mean; *** GPS Signal at lower 90% CI bound ≥2; **Signal at lower 95% CI bound >1.5; Table includes (1) all major disease category headings; (2) any disease categories that signaled for GPS or with a *p*-value ≤ 0.10; (3) any parents/grandparent of categories that signaled, and (4) any sibling of a disease category that signaled as long as there were observed events. We excluded any categories that were exactly the same as the parent.

### 3.3. Overall Signaling Comparison

Table 4 presents all 71 signals. TreeScan identified 71 signals, 49 at the highest threshold (*p* ≤ 0.001) and 22 at medium threshold (0.001< *p* < 0.05). GPS identified 48 signals; all high threshold GPS signals were also high threshold TreeScan signals, and 84% (21/25) of the moderate threshold GPS signals were high TreeScan signals. There were five high threshold TreeScan signals that did not signal for GPS, all had low O/E ratios (1.4 to 1.9) and large observed counts (67 to 187). 

**Table 4:** Comparison of all GPS and TreeScan signals across the four drugs studied by threshold.

	Tree-Based Scan Statistic (TreeScan) Signal Thresholds		
**GPS Signal Thresholds**	*p* ≤ 0.001	0.001< *p* ≤ 0.05	Total (%)
High	23	0	23 (32)
Medium	21	4	25 (35)
No signal	5	18	23 (32)
TOTAL	49	22	71
**GPS Thresholds**			
Medium: lower 95% CI bound ≥1.5			
High: lower 90% CI bound >2.0			
Thresholds are mutually exclusive			

Note: Data are no. (% of all signals). GPS: Gamma Poisson Shrinker.

## 4. Discussion

Electronic healthcare databases hold promise for pharmacovigilance because they address common shortcomings inherent to spontaneous reporting systems, offer large sample sizes and the potential to study subgroups, and include longitudinal medical information for a defined population. This is the first study to apply the GPS to population-based observational data in a multi-site environment for assessment of non-prespecified outcomes. Prior studies have implemented GPS in similar environments but have focused on pre-specified drug-event pairs. 

We identified 71 signals across four drug products. Of the 48 GPS signals identified, 23 (48%) signaled at the highest threshold. We did not formally evaluate each signal for clinical plausibility or prior knowledge of association, or prioritize them for refinement. All GPS signals were either known associations or could be reasonably attributed to confounding. We counted as unique every signal at each level of the hierarchical tree, an approach that overestimates the number of signals that would require refinement under a real-world implementation. For example, we counted signals for Diseases of the Nervous System and Sense Organs (06), Eye Disorders (06.07), Cataracts (06.07.01), and Glaucoma (06.07.03) as four distinct signals, although the higher-level signals (06 and 06.07) were almost entirely made up of the two lower-level signals and would not require four distinct signal refinement activities. 

The GPS and TreeScan results were similar with respect to the clinical areas that signaled, although findings varied by signaling threshold. In the few cases that GPS did not signal and TreeScan signaled at highest threshold, observed counts were high and O/E was low. This is expected behavior based on the nature of the two methods.

We note that using observational data for signal detection requires complex and often subjective analytic specifications, often unstated, that can directly affect the interpretation of the findings. Our implementation applied common epidemiologic approaches such as allowing individuals to contribute exposed and unexposed person time, and most importantly, involved identification of non-specified incident outcomes. The pilot was conducted using a fully distributed approach that did not require sharing of person-level protected health data but allowed identical definitions and analyses to be performed at each data partner. 

Implementation required specifications such as the baseline period, the allowable enrollment gap, the definition of contributed, exposed, and unexposed time, the creation of treatment episodes, allowable treatment gaps, right censoring decisions, and incident outcome definitions. A longer baseline period could reduce confounding by indication by identifying more patients with prevalent comorbid conditions, but would reduce overall cohort size due to lack of complete baseline. The incident outcome also can be defined several ways taking into account baseline period, care setting (inpatient *versus* outpatient), and diagnosis coding hierarchy. By excluding diagnoses unlikely to be associated with an acute outcome we eliminated the possibility of identifying the excluded diagnoses as outcomes. Our exclusion of injury and poisoning codes is debatable; those codes could be valuable in identifying those risks, but there is some uncertainty about how well those codes reflect adverse events *versus* misuse of safe medications (e.g., overdose). Regardless, they could be added in future implementations, especially in combination with loosening our simplifying restriction that allowed only one incident event per person. Finally, we compared exposed to unexposed time, controlling for age group, sex, and health plan. Others options for confounding adjustment include using an exposed comparison cohort, narrower age stratifications, and matching or disease severity stratification. 

Further, we note that we did not identify a set of expected signals that we hoped to find (e.g., expected adverse drug events) or avoid (e.g., confounded signals). Rather, since others have identified GPS as a potentially valuable tool for signal detection, our goal was to investigate the feasibility of implementing GPS and TreeScan in a real-world large-scale data mining application using longitudinal electronic health data without limiting the analysis to pre-specified relationships. We reviewed the product label, medical literature, and consulted clinical experts (co-authors and others) to informally assess whether identified signals were known or reasonably expected due to confounding. In our view there is no clear and well defined list of all known adverse drug events. Most drug labels have extensive adverse event list, but there is no conclusive evidence that all these “adverse events” are caused by the drug. For black box warnings there is usually strong evidence, but most known adverse events do not generate a black box warning. Developing a comprehensive list of all possible adverse events was beyond the scope of the paper and inconsistent with our primary aim to assess implementation in a real world setting. 

There is no “correct” decision regarding signaling thresholds, only generally understood trade-offs between identifying more or fewer signals and the resulting changes in numbers of signals needing refinement. The ability to interpret the signal detection findings within the larger pharmacovigilance framework, the ability of the findings to inform decision-making, and effort needed to evaluate them are important factors in whether or not a surveillance approach is viable. For instance, a viable approach will generate signals with enough informational value that they can be quickly adjudicated as likely due to confounding, known, expected, or otherwise uninteresting *versus* those that require further evaluation and refinement. 

We have shown that the GPS method can be successfully applied to population-based health plan data and that it performs adequately, with the ability to detect known adverse events. Although the GPS always shrinks the O/E estimate to some value to reduce variability, it is not always the case that the O/E shrinks towards 1.0 [38]. We observed shrinkage towards approximately 1.5. This property could be seen as less than desirable. It reflects that across all the outcome events under study, there is an excess number of events compared to the expected. Thus, the O/E is shrunk towards some average O/E taken over all outcomes. The fact that this is more than 1.0 could be because the drug causes a whole range of adverse events, or more likely, that the drug is taken by a generally sicker or more frail population that experiences a whole range of comorbidities. Prior implementations of GPS in longitudinal data did not report this finding or any metrics regarding the prior distributions. This finding emphasizes the need for research with empirical Bayesian approaches to report details of the prior distribution so that it is clear to which value shrinkage occurs, and in general the importance of transparency of reported findings, around for example, signaling thresholds and the need to consider alternative implementation approaches such as the use of zero-inflated Poisson to account for the many empty cells and underlying variability. More research regarding the appropriate implementation strategy for GPS using longitudinal data and signaling thresholds and strategies for multi-level testing are also needed, including the potential to adjust thresholds and approaches based on the specific surveillance target(s) and perhaps observed prior distributions.

Compared with TreeScan, there are both strengths and weaknesses to GPS, and it may be ideal to employ both methods simultaneously, using the combined results to better strengthen, refute, and understand signals. A strength of the GPS method is the Bayesian probability intervals; shrinkage of the point estimates towards the population average of O/E is a possible strength, although care must be taken to insure “shrinkage” towards values over unity does not introduce signals. We suggest future implementations carefully assess the GPS parameter sets to understand the distribution of O/E in the underlying population. A major strength of the TreeScan is the formal adjustment for multiple testing and the ability to analyze different levels of disease granularity in a single combined analysis. 

Signal detection is one step in the continuum of medical product safety surveillance. Approaches that generate statistical signals difficult to refine are of little practical use [46]—potentially creating an unfortunate situation in which new signal detection methods generate more heat than light. Therefore, it is critical that a robust medical product safety surveillance system have efficient mechanisms—such as those proposed by diverse stakeholders including the governmental agencies, academia and the pharmaceutical industry to quickly prioritize, refine and evaluate and act on signals. Examples of novel approaches to surveillance and signal management include the work of the US FDA Sentinel System [31,47,48] the WHO Collaborating Centre for International Drug Monitoring [49], the Asian Pharmacoepidemiology Network [50], and the European Network of Centres for Pharmacoepidemiology and Pharmacovigilance [51], and are being tested in multiple major international initiatives [52,53,54]. 
